# Announcement: The Third International Conference of Gold and Silver in Medicine

**DOI:** 10.1155/MBD.1994.338

**Published:** 1994


					l/ol. 1, No. 4, 1994 Announcements
THE THIRD INTERNATIONAL CONFERENCE OF
GOLD AND SILVER IN MEDICINE
The Third International Conference on Gold and Silver in Medicine
will be held next summer in Milwaukee Wisconsin USA, August 18-20,
1994.
The site of the meeting will the University of Wisconsin, Milwaukee.
Accomodations will be available in student dormitories at UWM Sandburg
Residence Hall and also at the Milwaukee Hilton Hotel.
Abstracts of Meeting talks will be published. The deadline for receipt is March
30, 1994.
Tentative registration fees have been set as $150 for participants, $100 for
postdoctoral associates and $75 for students. We hope to be able to provide some
level of assistance, especially to students and travelers from outside North
America.
The current list of invited speakers includes:
Richard Beard, Drexel University
Richard Elder, University of Cincinnati
Ernst Gleichman, Heinrich-Heine University, Dusseldorf
Colin Lock, McMaster University, Ontario, Canada
Peter Sadler, Birkbeck College, University of London, UK
Frank Shaw, University of Wisconsin-Milwaukee
Ewen Smith, Strathclyde University, Glascow, UK
Martin Stillman, University of Western Ontario, Canada
Katherine Tepperman, University of Cincinnati
If you wish further information contact: "Richard.Eider@ UC.EDU" via INTERNET
or FAX (513) 556-9:239 or voice (513) 556-9:200.
338

				

